# Mitochondrial Population in Mouse Eosinophils: Ultrastructural Dynamics in Cell Differentiation and Inflammatory Diseases

**DOI:** 10.3389/fcell.2022.836755

**Published:** 2022-03-21

**Authors:** Kennedy Bonjour, Cinthia Palazzi, Thiago P. Silva, Kássia K. Malta, Vitor H. Neves, Eliane G. Oliveira-Barros, Igor Neves, Victor A. Kersten, Bruno T. Fortuna, Amali E. Samarasinghe, Peter F. Weller, Christianne Bandeira-Melo, Rossana C. N. Melo

**Affiliations:** ^1^ Laboratory of Cellular Biology, Department of Biology, ICB, Federal University of Juiz de Fora, Rua José Lourenço Kelmer, Juiz de Fora, Brazil; ^2^ Laboratory of Inflammation, Institute of Biophysics Carlos Chagas Filho, Federal University of Rio de Janeiro, Rio de Janeiro, Brazil; ^3^ Division of Pulmonology, Allergy-Immunology and Sleep, Department of Pediatrics, College of Medicine, University of Tennessee Health Science Center, Memphis, TN, United States; ^4^ Department of Medicine, Beth Israel Deaconess Medical Center, Harvard Medical School, Boston, MA, United States

**Keywords:** eosinophils, mouse, mitochondrial dynamics, mitochondrial architecture, mitochondria ultrastructure, electron tomography, eosinophilopoiesis, mitochondria contact sites

## Abstract

Mitochondria are multifunctional organelles of which ultrastructure is tightly linked to cell physiology. Accumulating evidence shows that mitochondrial remodeling has an impact on immune responses, but our current understanding of the mitochondrial architecture, interactions, and morphological changes in immune cells, mainly in eosinophils, is still poorly known. Here, we applied transmission electron microscopy (TEM), single-cell imaging analysis, and electron tomography, a technique that provides three-dimensional (3D) views at high resolution, to investigate mitochondrial dynamics in mouse eosinophils developing in cultures as well as in the context of inflammatory diseases characterized by recruitment and activation of these cells (mouse models of asthma, H1N1 influenza A virus (IAV) infection, and schistosomiasis mansoni). First, quantitative analyses showed that the mitochondrial area decrease 70% during eosinophil development (from undifferentiated precursor cells to mature eosinophils). Mitophagy, a consistent process revealed by TEM in immature but not in mature eosinophils, is likely operating in mitochondrial clearance during eosinophilopoiesis. Events of mitochondria interaction (inter-organelle membrane contacts) were also detected and quantitated within developing eosinophils and included mitochondria-endoplasmic reticulum, mitochondria-mitochondria, and mitochondria-secretory granules, all of them significantly higher in numbers in immature compared to mature cells. Moreover, single-mitochondrion analyses revealed that as the eosinophil matures, mitochondria cristae significantly increase in number and reshape to lamellar morphology. Eosinophils did not change (asthma) or reduced (IAV and Schistosoma infections) their mitochondrial mass in response to inflammatory diseases. However, asthma and schistosomiasis, but not IAV infection, induced amplification of both cristae numbers and volume in individual mitochondria. Mitochondrial cristae remodeling occurred in all inflammatory conditions with the proportions of mitochondria containing only lamellar or tubular, or mixed cristae (an ultrastructural aspect seen just in tissue eosinophils) depending on the tissue/disease microenvironment. The ability of mitochondria to interact with granules, mainly mobilized ones, was remarkably captured by TEM in eosinophils participating in all inflammatory diseases. Altogether, we demonstrate that the processes of eosinophilopoiesis and inflammation-induced activation interfere with the mitochondrial dynamics within mouse eosinophils leading to cristae remodeling and inter-organelle contacts. The understanding of how mitochondrial dynamics contribute to eosinophil immune functions is an open interesting field to be explored.

## Introduction

Mitochondria are multifaceted organelles with an architecture extraordinarily interconnected with cellular functions. Mitochondria can change their structure in coordination with both physiological and pathological cellular processes and these alterations influence a plethora of cell signaling pathways such as bioenergetics (Galloway et al., 2012; Bahat and Gross, 2019) and cell fate (Bahat and Gross, 2019). Mitochondria are fascinating organelles considering that they combine high plasticity with continuous cycles of membrane fusion and fission and remarkable ability to establish membrane contacts with other organelles and to work as a cooperative population [reviewed in (Galloway et al., 2012; Chen and Chan, 2017)]. The term mitochondrial dynamics is used to describe structural events of mitochondrial remodeling involving size, shape, activity, trafficking, and inter-organelle interactions within the cell cytoplasm (Cervantes-Silva et al., 2021).

The impact of mitochondrial dynamics in cells from the immune system and in the modulation of immune responses has been addressed (reviewed in (Campello and Scorrano, 2010; Angajala et al., 2018; Cervantes-Silva et al., 2021). For example, T cell activation induces cristae remodeling that regulates metabolism (Buck et al., 2016). Other aspects of the mitochondrial dynamics such as elongation and mitochondria-ER tethering have been recognized as important events related to the innate immune system functional activities (Campello and Scorrano, 2010; Angajala et al., 2018; Cervantes-Silva et al., 2021). However, our current understanding of the mitochondrial architecture, interactions, and morphological changes in immune cells during their development and under homeostatic and pathological conditions is still poorly known, especially in eosinophils.

Our group has been studying ultrastructural mechanisms underlying the functions of eosinophils, cells from the innate immune system, in health and in a wide variety of diseases, both in humans and mouse models [reviewed in (Melo et al., 2008; Melo et al., 2010; Melo and Weller, 2016, 2018; Melo et al., 2022). Here, we applied transmission electron microscopy (TEM), single-cell imaging analysis, and electron tomography, a technique that provides three-dimensional (3D) views at high resolution, to investigate mitochondrial dynamics in mouse eosinophils developing in cultures as well as in the context of inflammatory diseases characterized by recruitment and activation of these cells (mouse models of asthma, H1N1 influenza A virus (IAV), and schistosomiasis mansoni infections). Our findings show that mitochondrial remodeling and inter-organelle interactions, including an undescribed contact with secretory granules, are closely associated with both eosinophilopoiesis and eosinophil inflammatory responses.

## Material and Methods

### Ethics Statement

This study was carried out in accordance with protocols and guidelines approved by the Animal Care and Use Committees of the following Institutions: Federal University of Rio de Janeiro, Brazil (CEUA-CCS/UFRJ 090/18); Oswaldo Cruz Foundation, Brazil (CEUA/Fiocruz-0213-4/2007; CEUA/Fiocruz 32/2012); and University of Tennessee Health Science Center (UTHSC), United States of America.

### Animals

Six-week-old female BALB/c and Swiss Webster mice were obtained from the animal facilities of the Institute of Biophysics Carlos Chagas Filho (UFRJ, Rio de Janeiro), and Oswaldo Cruz Foundation breeding unit (CECAL/Fiocruz, Rio de Janeiro). Six-week-old female C57BL/6J mice were purchased from Jackson Laboratories (Bar Harbor, ME). Mice were housed in environmentally controlled conditions (22°C, a 12 h light/dark cycle with the light cycle from 6 a.m. to 6 p.m. and the dark cycle from 6 p.m. to 6 a.m.) and monitored daily for survival and wellbeing status (home cage evaluation, body condition, skin lesions, mobility, and other general conditions). Animals were housed at least 1 week before investigation and all experiments were performed during the light cycle.

### Animal Models of Diseases

#### Asthma Model

BALB/c mice, weighing 20-25 g each and with *ad libitum* access to standard ovabulmin (OVA)-free laboratory chow and water, were immunized subcutaneously on day 0 with 50 μg OVA (grade V; Sigma-Aldrich, St Louis, MO, USA) and 5 μg aluminum hydroxide (Sigma) in 200 μL of 0.9% NaCl (sterile saline) as before (Serra et al., 2012). On day 14 after injection, mice were boosted with the same solution above administered intraperitoneally and intranasal OVA challenges (25 μg/25 μL in saline) were administrated on the subsequent 4 weeks (3 times per week) under halothane volatile anesthesia. Sensitized control mice were challenged only with vehicle. Thirty days after the last instillation, animals (*n* = 5) were euthanized in a CO_2_ chamber, perfused with 20 ml of sterile PBS to remove blood, and fragments of the right lung lobe immediately fixed and processed for both histology (Nandedkar et al., 2008) and TEM (detailed below). Resident lung eosinophils isolated from nontreated mice (*n* = 5) as before (Percopo et al., 2017) were used as controls. Histopathological analyses confirmed the extensive deposition of collagen fibers and eosinophilic inflammatory infiltrate typical of allergic inflammation (Supplementary Figure S1).

#### Influenza Model

Weight-matched C57BL/6J mice (*n* = 5) were infected with 1000 TCID_50_ pH1N1 virus, intranasally under isoflurane anesthesia as previously described (Samarasinghe et al., 2017). The A/CA/04/2009 strain (pH1N1) is a clinical IAV isolate recovered during the 2009 IAV pandemic, kindly provided by Dr. Richard Webby (SJCRH) and propagated in Madin-Darby canine kidney (MDCK) cells (ATCC, Manassas, VA, USA). Strains of pH1N1 were sequenced to confirm genetic integrity of hemagglutinin and neuraminidase genes and stored as single use aliquots at −80°C. The concentration of virus was determined as described (LeMessurier et al., 2020). The term “flu” is used to refer to mice that develop influenza as a consequence of infection with pH1N1. Mice were weighed daily starting before infection and euthanized by CO_2_ asphyxiation at 7 days post infection. The infectious dose induced morbidity but no mortality. In the noninfected control group (*n* = 5), mice received just saline. Bronchoalveolar lavage (BAL) was performed by 2 washes each with 1 ml PBS and recovered cells immediately fixed and processed for TEM, as detailed below.

#### Schistosomiasis Mansoni Model

Swiss Webster mice aged 70 days were injected subcutaneously with a single inoculum of cercariae of *S. mansoni* (100 cercariae/mouse), LE strain as previous work (Dias et al., 2018). Infection was confirmed by findings of parasite eggs in the rodent feces at week five of infection (Hoffman et al., 1934). Infected animals (*n* = 6) and respective uninfected controls (*n* = 6) from the same age were euthanized at 55 days of infection (acute phase) (Borojevic et al., 1981) by exsanguination (full bleed) under deep anesthesia by cardiac puncture. The anesthetic protocols included ketamine (100 mg/ml) combined with acepromazine (10 mg/ml) at a ratio of 9:1 (dose of 0.15 ml/100 g body weight) (Hawk et al., 2005). Fragments of the large intestine (from infected and noninfected controls) were immediately fixed and processed for both histology as previous work (Amaral et al., 2017) and TEM (detailed below). Histopathological analyses confirmed the development of a typical granulomatous inflammatory response around parasite eggs in the large intestine of *Schistosoma mansoni*-infected mice (Supplementary Figure S1) as previously detailed by our group (Amaral et al., 2017).

### Cultures of Bone Marrow-Derived Eosinophils

Bone marrow–derived eosinophils (BMdEos) were generated as described (Dyer et al., 2008) from the tibias and femurs of female BALB/c mice. Each culture was prepared through undifferentiated cells from two animals and a total of 21 cultures were used in the present study. Cells were collected and evaluated at days 0, 4, 6, 8, 10, 12 and 14, with cell collections from three different cultures for each point for experiments by light microscopy (cytospin preparations stained with routine hematological stains), flow cytometry, and transmission electron microscopy (TEM) as described below. Cell viability was >93% as determined by the trypan blue exclusion test.

### Flow Cytometry

BMdEos (1 × 10^6^ cells/well) were incubated with fluorescently tagged antibodies in PBS with 0.1% BSA for 30 min at 4°C. After staining, the cells were washed with PBS with 0.1% BSA and fixed in 4% paraformaldehyde. Data were acquired in a FACS Calibur flow cytometer (BD Biosciences) and analyzed using FloJo software v 7.1 (Tree Star, Ashland, OR). Positive cells were identified by comparison to the appropriately conjugated isotype control. Antibodies (all from BD Pharmingen) used included PE-conjugated rat anti-mouse Siglec-F or PE-conjugated IgG2a isotype control.

### TEM

Tissue fragments (lungs and intestines) were fixed in a mixture of freshly prepared aldehydes [final concentration of 1% paraformaldehyde and 2.5% glutaraldehyde (EM grade, 50% aqueous, Electron Microscopy Sciences-EMS, Hatfield, PA)] in 0.1 M sodium phosphate buffer, pH 7.4, at room temperature (RT). After 2h, fragments were sliced into small pieces of 1 mm^3^ and fixed in the same fixative at 4°C. Next, the fragments were washed twice (4 h each) in 0.1 M sodium phosphate buffer in 0.1 M sodium phosphate buffer at 4°C and kept in the same buffer for additional processing. Cell suspensions (samples from cultures, BALs, and resident eosinophils isolated from control lungs) were fixed in the same fixative solution as above (while still in suspension) for 1 h at RT or in 1% paraformaldehyde and 1.5% glutaraldehyde) in 1 M sodium cacodylate buffer, pH 7.4, for 1 h at RT and embedded in agar pellets for accurate handling of the eosinophils as previously described (Melo et al., 2005). All samples were post-fixed in 1% osmium tetroxide in Sym-Collidine buffer, pH 7.4, for 2 h at RT. After washing with sodium maleate buffer, pH 5.2, they were stained *en bloc* in 2% uranyl acetate (EMS) in 0.05 M sodium maleate buffer, pH 6.0, for 2 h at RT and washed in the same buffer as before prior to dehydration in graded ethanol and acetone, and infiltration and embedding with a propylene oxide-Epon sequence (Eponate 12 Resin, Ted Pella, Redding, CA, USA). After polymerization at 60°C for 16 h, thin sections were obtained using a diamond knife on an ultramicrotome (Leica, Bannockburn, IL). Sections were mounted on uncoated 200-mesh copper grids (Ted Pella) before staining with lead citrate and viewed with a transmission electron microscope (CM 10; Philips, or Tecnai G2-20-ThermoFischer Scientific/FEI 2006, Eindhoven, the Netherlands) at 80-120 KV.

### TEM Quantitative Analyses

Electron micrographs showing the entire cell profile, nucleus, and intact plasma membrane were randomly taken from eosinophils during different conditions/diseases. The following aspects were evaluated and quantitated in the eosinophil cytoplasm (total *n* = 204 cells and 1720 mitochondria): 1) Mitochondrial numbers; 2) Mitochondrial size (area of individual mitochondria in µm^2^); 3) Mitochondria proportion/cell section (total mitochondrial area/[cell area—nucleus area]) (Picard et al., 2013); 4) Total number of mitochondrial cristae per µm^2^ of mitochondrial area; 5) Cristae morphology; 7) Cristae volume (the sum of the areas of all the cristae in a single mitochondrion divided by the mitochondrion area) (Lam et al., 2021). Cristae shapes were classified in three morphological patterns: lamellar, tubular, or mixed. Lamellar cristae were identified in thin sections as parallel linear structures resembling the format of “shelves”. The tubular pattern of cristae was identified by the presence of “small circles” arranged in the mitochondrial matrix while the presence of lamellar and tubular cristae within a single mitochondrion denoted mitochondria with “mixed” cristae. Additionally, mitochondrial interactions were investigated in the cytoplasm. We quantitated all inter-organelle interactions involving mitochondria represented by membrane contacts and the number of these interactions was expressed per cell section.

### Electron Tomography

Electron tomography and 3D reconstruction were performed as previous works from our group (Melo et al., 2005; Melo et al., 2013). Eponate sections (200 nm-thickness) were collected on 100 mesh-formvar coated grids. Samples were contrasted with lead citrate and the tilt series acquired at 120 kV on a Tecnai Spirit G12 microscope (Thermo Fisher Scientific/FEI, Eindhoven, Netherlands) with an Eagle 4k x 4k camera (Thermo Fisher Scientific/FEI) using SerialEM software (Mastronarde, 2005). Images in a tilt series were collected at intervals of -65° to +65° in 1° intervals using a mean of 1s exposure per micrograph. Tomograms were generated using the SerialEM software. All tilted images were aligned to a common tilting axis using cross correlation, and the volumes were reconstructed by real-space weighted rear projection. A total of 7 tomograms were analyzed to characterize the mitochondrial cristae patterns and other morphological aspects. Modeling was carried out using IMOD software (Mastronarde and Held, 2017).

### Statistical Analyses

One-way or 2-way ANOVA followed by Tukey multiple comparisons test or the Student’s t test was performed using GraphPad Prism version 6.01 (GraphPad Software, San Diego, CA, USA). The normal distribution analysis (Shapiro-Wilk test) was also used to evaluate the shape descriptors. *p* value <0.05 was considered statistically significant. All quantitative analyses were performed using Fiji software (National Institutes of Health, Bethesda, MD, USA).

Multivariate logistic regression analyses were additionally performed using SPSS data analysis statistics software system version 20.0 (SPSS Inc., Chicago, IL, USA) to estimate the probability (or risk) of a particular outcome (immature or mature eosinophils) given the values of the independent variables, that is, the effect of a group of independent variables on a binary outcome by quantifying each independent variable’s unique contribution (Liou et al., 2012; Schober and Vetter, 2021). The results of these logistic regression models are presented as the odds ratio (OR). The *p* value <0.05 was considered statistically significant.

## Results

### Maturational Stages of Mouse Eosinophils Revealed by TEM

**FIGURE 1:**
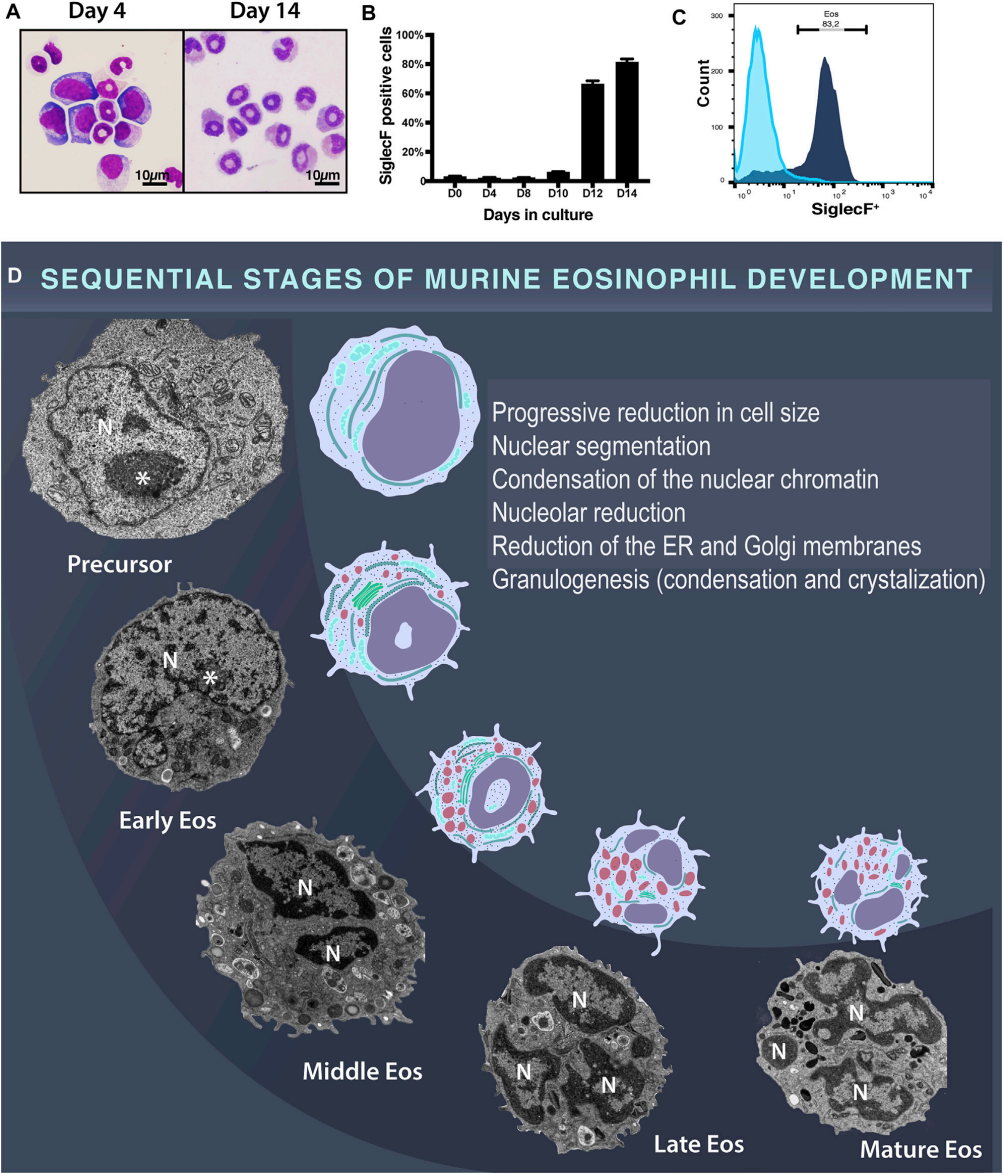
Mouse eosinophil development in *ex vivo* culture systems of BMdEos. **(A)** Developmental cells seen under light microscopy after routine staining. Note the ring-shaped eosinophil nuclei. **(B,C)** Flow cytometric analysis of Siglec F expression in 3 independent mouse cultures. **(D)** Developmental eosinophil stages observed by TEM. In the early stage of development, eosinophils are characterized by a large, eccentric, and round nucleus with a prominent nucleolus (*), which undergo progressive lobulation. Nuclear changes occur in parallel with a reduction in cell size and in the amount of the endoplasmic reticulum and Golgi membranes. Granulogenesis involves content filling, condensation, and crystallization. N, nucleus.

First, an *ex vivo* culture system, which generates large numbers of eosinophils at high purity was established from unselected bone marrow progenitors as described (Dyer et al., 2008). Cells were evaluated at days 0, 4, 6, 8, 10, 12, and 14 by light microscopy and flow cytometry. Cytospin preparations showed cells with different nuclear morphologies including ring-shaped nuclei typical of mouse eosinophils when seen under light microscopy (Figure 1A)(Lee et al., 2012). By days 12 and 14, the proportion of cells expressing Siglec F, an eosinophil surface receptor considered a marker for this cell (Dyer et al., 2008), was 66.6 and 83.0%, respectively (Figures 1B,C).

Second, eosinophils developing in cultures as above were processed for TEM using a protocol that enables fixation of the cells while still in suspension (before any centrifugation/cell manipulation) for optimal morphological preservation (Melo et al., 2005). Considering that eosinophilopoiesis is a continuum of gradual morphologic changes, which lead to the formation of mature eosinophils, here we used the terms early, middle, late, and mature eosinophils to define the stages of eosinophil development when seen under the electron microscope (Melo et al., 2022) (Figure 1D). These stages have also been referred to as early, middle, late, and mature eosinophilic myelocytes (Dvorak et al., 1991; Melo et al., 2022).

An important feature of the eosinophil developmental morphology is related to the nucleus, which is initially round, large, and predominantly euchromatic becoming segmented and with condensed peripheral chromatin (Figure 1D). Another distinctive eosinophilopoiesis-associated event is the formation of secretory granules (granulogenesis). Indeed, the earliest ultrastructural feature that characterizes cells of the eosinophilic lineage, thus distinguishing them from a common precursor (myeloblast-like), is the presence of large cytoplasmic granules (Scott and Horn, 1970). During maturation, these granules, termed specific or secretory granules, both in humans and rodents, undergo condensation and crystallization [reviewed in (Melo and Weller, 2018)]. Thus, based on previous observations in humans (Melo and Weller, 2018) and current study in mice, eosinophil granulogenesis involves the initial formation of a population of coreless granules (immature specific granules) that progresses to mature cored granules (specific granules). The presence of these cored granules containing a typical electron-dense crystalloid in the cytoplasm indicates a late stage of eosinophil development, which evolves to a completely mature eosinophil in which these granules predominate (Figure 1D).

By correlating the ultrastructural aspects of the cytoplasmic secretory granules and nucleus, we followed the development of mouse eosinophils in cultures, which was also accompanied by a reduction of the cell size (Figure 1D). From undifferentiated precursor to mature eosinophil, there was a reduction of 66.5% of the eosinophil size (cell areas in μm^2^ of 64.6 ± 3.8, 43.9 ± 2.7 and 28.2 ± 2.1 for precursor (myeloblast-like), immature, and mature eosinophils, respectively, n = total of 88 cells, mean ± SEM).

### Eosinophil Differentiation Leads to a Reduction of the Mitochondrial Population

**FIGURE 2:**
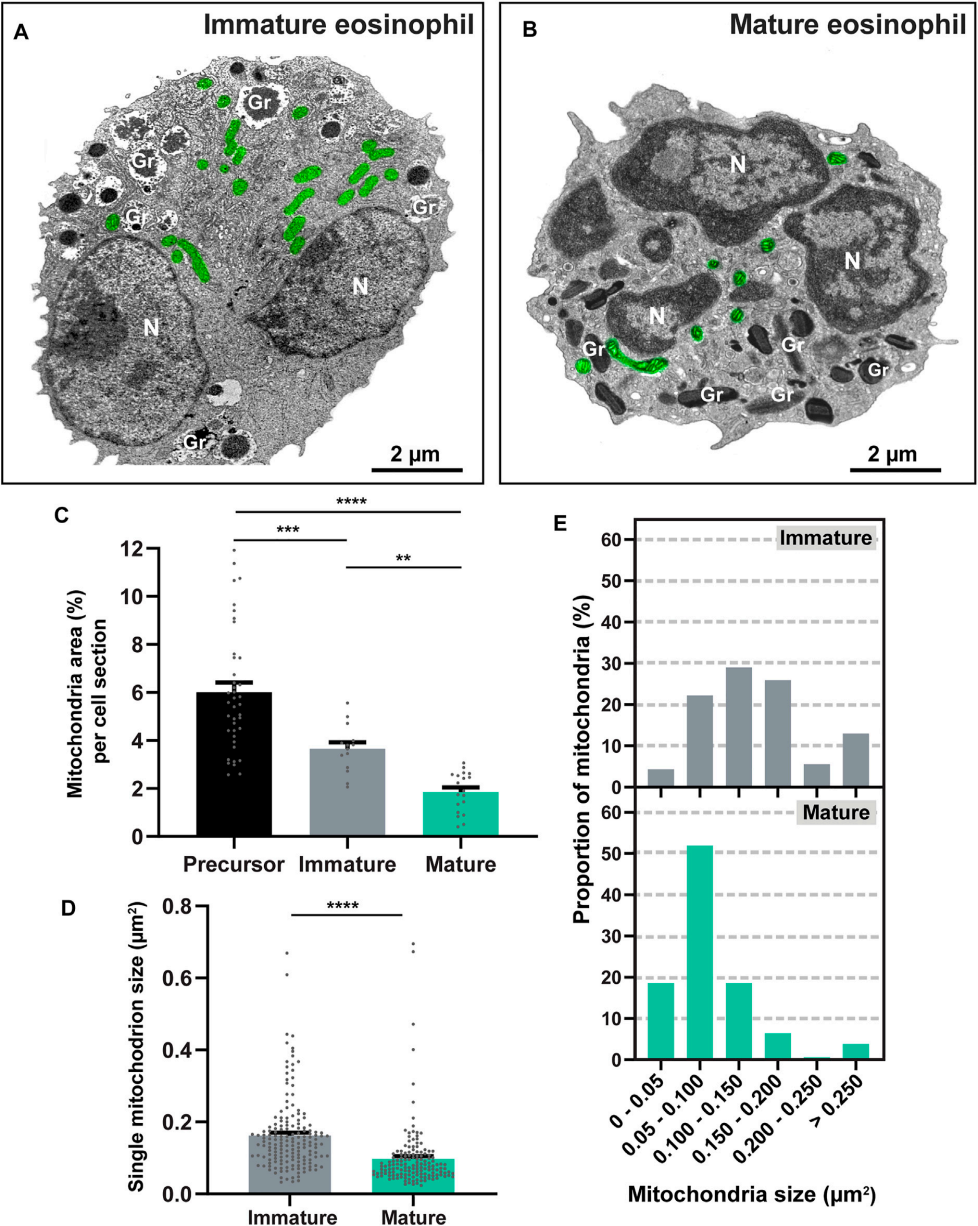
Mouse eosinophil maturation leads to the reduction of mitochondrial population. **(A,B)** Electron micrographs of a representative immature and mature eosinophil observed in a BMdEos culture. In the immature eosinophil, note round immature specific granules (Gr) in process of content filling and a nucleus (N), already segmented but still in process of chromatin condensation. In the mature eosinophil, specific granules (Gr) are elongated with typical internal crystalloid and nucleus (N) exhibit clear euchromatin-heterochromatin distinction. Note the mitochondrial population (pseudocolored green). **(C–E)** The mitochondrial area in the cytoplasm and the size of individual mitochondria significantly reduce during eosinophilopoiesis. Samples were prepared for TEM and quantitative analyses performed using Fiji software. A total of 925 mitochondria were evaluated at high resolution within 88 cells collected from at least three independent cell cultures. Scattered dots in **(C,D)** represent single mitochondria data. Results are expressed as means ± SEM (**p < 0.01; ***p < 0.001; ****p < 0.0001).

Having established the maturational stages of mouse eosinophils (Figure 1D), we next focused on the study of the mitochondrial population at high resolution within the cytoplasm of these cells. Eosinophils exhibiting an early and middle stage of development, that is, with most of their granules seen as coreless granules, were considered with a profile predominantly immature while eosinophils containing at least 60% of their total granule population as cored granules (late and mature stages of development) were deemed as having a profile predominantly mature (Melo et al., 2022). Thus, several morphological aspects of the mitochondrial population were evaluated and compared between these two groups of immature and mature eosinophils (Figures 2A,B).

First, we questioned if the numbers of mitochondria would vary during the process of eosinophil maturation. Mitochondria were then quantitated by TEM in precursor cells, immature and mature eosinophils collected from cultures on different days of establishment as above. By evaluating a total of 88 electron micrographs of eosinophils showing the entire cell profile and nucleus and a total of 925 mitochondria (*n* = 608 from precursor cells; *n* = 166 from immature eosinophils; and *n* = 151 from mature eosinophils), we found that the range of mitochondria numbers per cell section was 5-32, 4-26, and 2-20 in precursor cells, immature, and mature eosinophils, respectively. However, because mitochondria can display different sizes varying from round to very elongated morphology, we consider that the evaluation of the area occupied by these organelles in the eosinophil cytoplasm would be a more accurate parameter for mitochondria quantification than simply enumerating these organelles. Thus, by performing imaging analyses, the mitochondrial area (µm^2^) was delimited and measured in relation to the cytoplasmic area in each eosinophil section (Figure 2C). Quantitative TEM showed that eosinophilopoiesis led to a reduction of 70% in the mitochondrial area (from precursor to mature cells (precursor: 6.01 ± 0.4%, *n* = 608 mitochondria evaluated within 40 cells versus mature: 1.80 ± 0.19%, *n* = 151 mitochondria evaluated within 20 cells; mean ± SEM, *p* < 0.0001) (Figures 2A–C). When eosinophils with immature and mature profiles were compared, we found that maturation led to a reduction of ∼50% in the mitochondrial area (immature: 3.65 ± 0.26, *n* = 166 mitochondria evaluated within 28 cells versus mature: 1.80 ± 0.19%, *n* = 151 mitochondria evaluated within 20 cells), mean ± SEM; *p* < 0.01) (Figures 2A–C).

We also analyzed the range of mitochondrial sizes at single level (area of individual mitochondria in thin sections) (Figures 2D,E). In immature cells, the mean size of a mitochondrion was 0.162 ± 0.007 µm^2^ (mean ± SEM, *n* = 166 mitochondria), while mature cells had a mean size of 0.098 ± 0.007 µm^2^ (mean ± SEM, *n* = 151 mitochondria). As expected, a diversity of mitochondrial sizes was obtained. However, this range was much higher in immature [14–886 nm^2^ (*n* = 166 mitochondria)] compared to mature [0.019–0.380 µm^2^ (*n* = 151 mitochondria); *p* < 0.0001]. Interestingly, while most mitochondria (∼ 65%) in immature eosinophils had sizes higher than 0.100 µm^2^, mature eosinophils exhibited ∼ 70% of their mitochondrial population with sizes below 0.100 µm^2^ (Figure 2E).

### Mitochondrial Dynamics During Eosinophilopoiesis

Mitochondria are organelles with a high level of plasticity, able to undergo morphological changes and interact with other organelles consonant with the cell needs (Pernas and Scorrano, 2016). By examining a vast collection of mitochondria at high resolution within differentiating mouse eosinophils, we identified a spectrum of mitochondrial ultrastructural changes and mitochondria-associated events.

**FIGURE 3:**
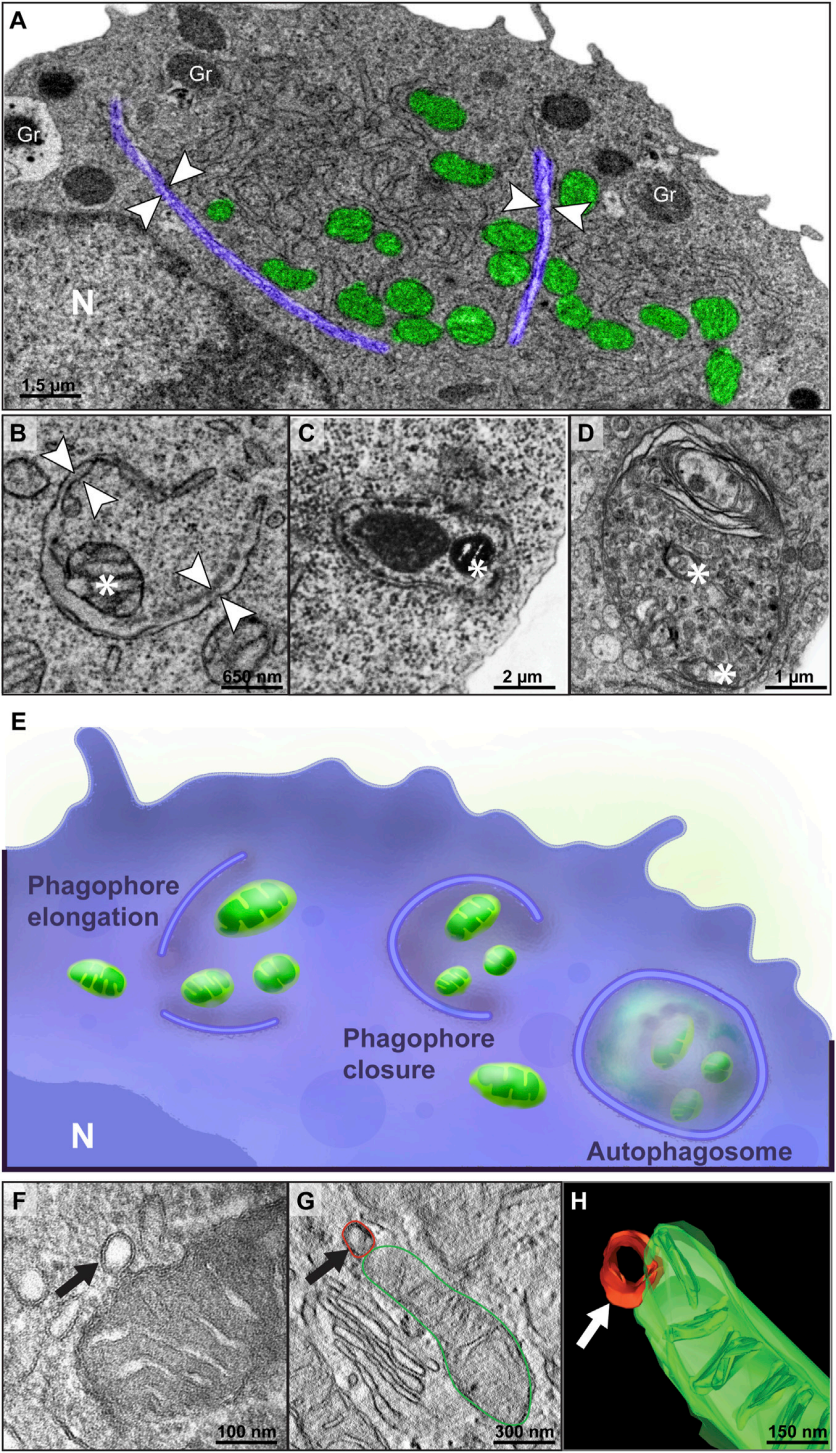
Mitophagy is a consistent process within immature eosinophils. **(A–D)** Mitophagy events represented by the formation of phagophore-like membranes (arrowheads) in different stages of extension enclosing single or groups of mitochondria and double-membrane vacuoles containing luminal mitochondria (*) **(C,D)** were observed by TEM. In **(A)**, note that the phagophore membranes (colored in purple) are segregating a portion of the cytoplasm containing mitochondria and ER cisternae. In **(E)**, an illustration shows the multi-step process of mitophagy. **(F–H)** Electron tomographic analyses captured a mitochondrial-derived vesicle (arrow) being formed from the outer membrane. Cells were prepared for conventional TEM and electron tomography as before (Melo et al., 2005). Gr, secretory granules; N, nucleus.

First, TEM revealed that mitophagy, the selective degradation of mitochondria by autophagy (Wang and Klionsky, 2011), was a consistent event during eosinophilopoiesis. Phagophore-like membranes, cup-shaped membranous structures, and autophagosomes, double-membrane-bound vacuoles, both canonical ultrastructural evidence for autophagy (Jung et al., 2019) were seen enclosing single and/or grouped mitochondria (Figures 3A–E and Supplementary Figure S2). While 15% of immature eosinophils showed ultrastructural evidence of mitophagy with 1-3 events indicative of this process per cell section (Figures 3A–E), eosinophils with a mature profile did not exhibit apparent mitophagy in the cytoplasm. Remarkably, application of electron tomography captured in 3D the occurrence of mitochondrial vesiculation, with mitochondrial-derived vesicles budding off the mitochondrial outer membrane (Figures 3F–H), a morphological event that can precedes mitophagy (McLelland et al., 2014; Sugiura et al., 2014). Thus, our findings identify different ultrastructural events of mitophagy, a mechanism that is likely operating in the reduction of the mitochondrial population during eosinophil development.

**FIGURE 4:**
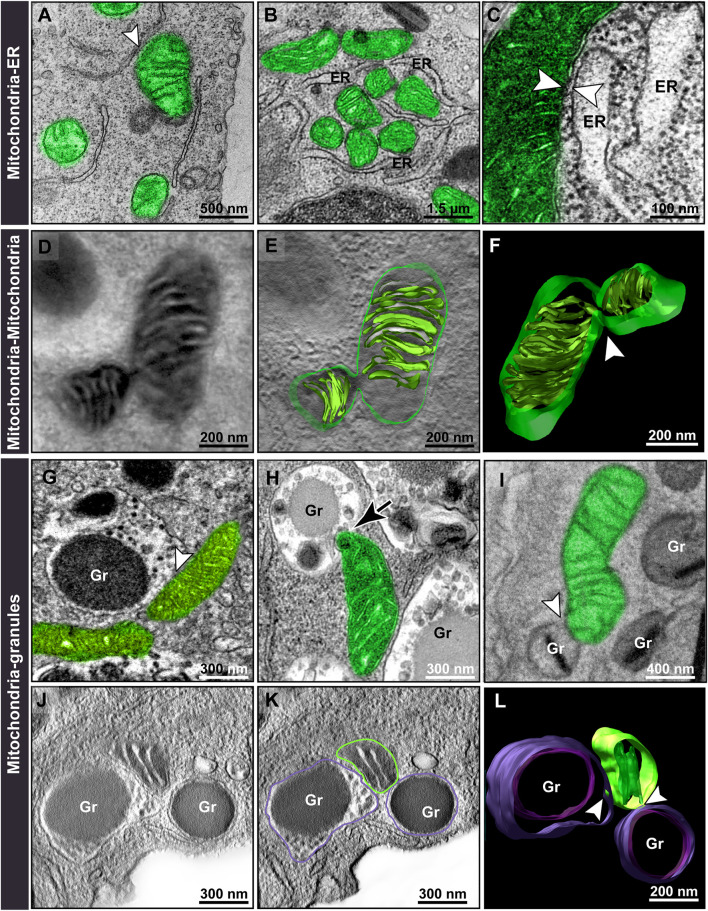
Mitochondrial interactions within immature and mature eosinophils. During eosinophilopoiesis, membrane contact sites (arrowheads) are observed between mitochondria and the endoplasmic reticulum (ER) **(A–C)**; other mitochondria **(D–F)**; and secretory granules **(G–L)**, mainly with coreless granules **(G,H)**. Note in **(H)**, a mitochondrion protruding into a coreless granule in process of content filling (arrow) while in **(I)**, a mitochondrion contacts a fully mature granule containing a typical electron-dense core (arrowhead). **(D–F)** and **(J–L)** show representative slices of tomograms and 3D models of mitochondria-mitochondria and mitochondria-granules **(J–L)** interactions, respectively. Virtual slices were extracted from 3D reconstructions of a 200 nm eosinophil section analyzed by fully automated electron tomography at 200 kV. Samples were prepared for conventional TEM and electron tomography as before (Melo et al., 2005). Mitochondria were pseudocolored green. Gr, specific granules.

Second, we identified different modes of mitochondrial interactions (inter-organelle membrane contacts) in the cytoplasm. In addition to the recognized mitochondria-ER (Figures 4A–C) and mitochondrion-mitochondrion association as seen here by electron tomography (Figures 4D–F), a remarkable and unexpected interaction of mitochondria with secretory granules was observed by both conventional TEM (Figures 4G–I) and electron tomography (Figures 4J–L). Interestingly, this granule-mitochondrion association occasionally was seen as mitochondrial protrusions into the granules (Figure 4H) and not merely as contact sites.

**FIGURE 5:**
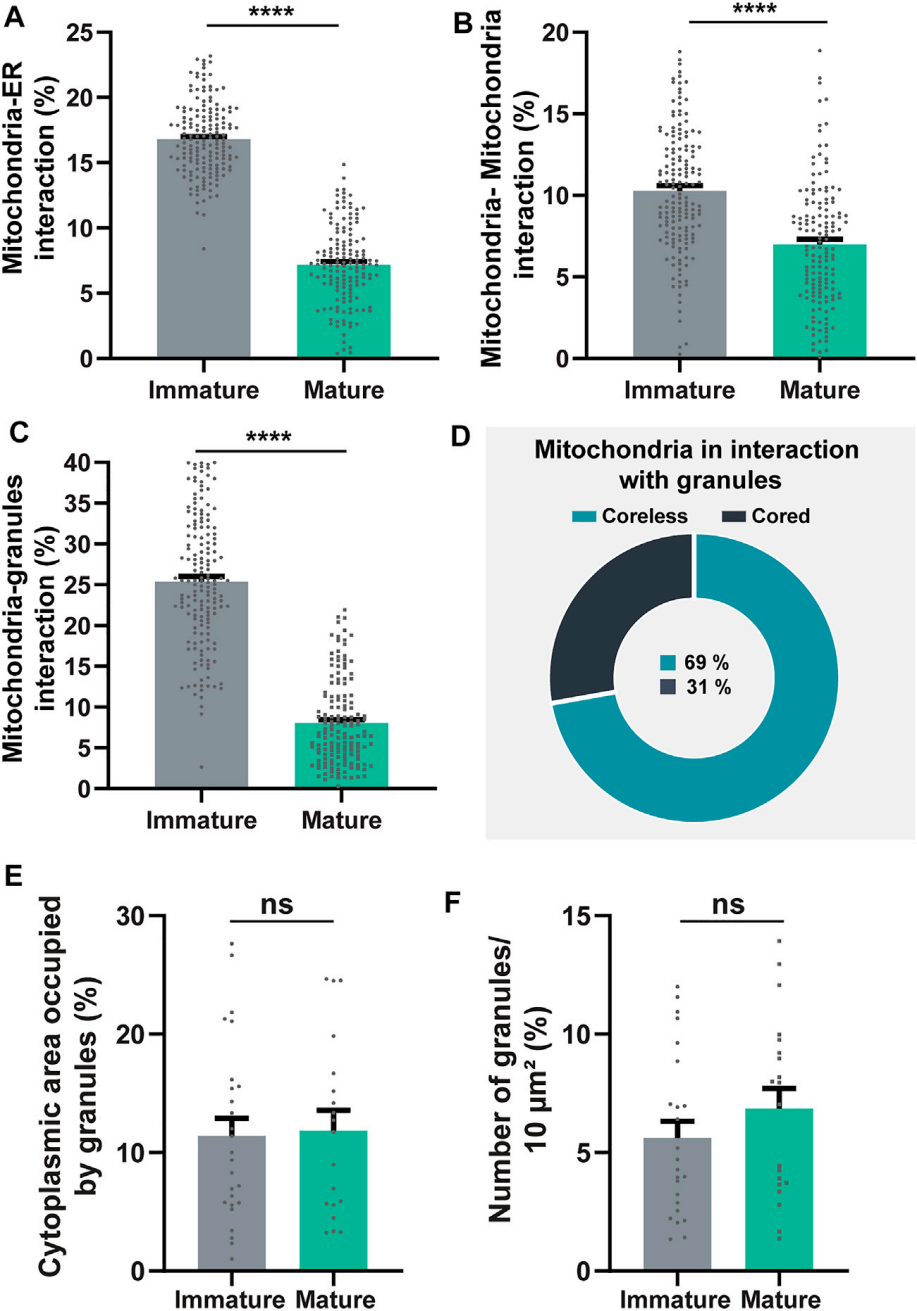
Quantitative analyses of mitochondria inter-organelle interactions and granule distribution in the cytoplasm during mouse eosinophil development. **(A–C)** Mitochondria-ER, mitochondria-mitochondria, and mitochondria-granules associations (membrane contact sites) are significantly higher within immature compared to mature eosinophils. **(D)** In immature eosinophils, mitochondria contact predominantly coreless granules. **(E,F)** The cytoplasmic area occupied by secretory granules does not change in immature compared with mature eosinophils. Scattered dots in A-C represent single mitochondria while in E and F scattered dots represent single-cell sections. A total of 317 mitochondria (166 from immature and 151 from mature cells) and 875 granules (521 from immature and 354 from mature cells) were evaluated at high resolution in 48 eosinophils (28 immature and 20 mature) collected from at least three independent cell cultures. Results are expressed as means ± SEM (****p < 0.0001; ns (not significant) *p* > 0.05).

Quantitative TEM evaluating the number of mitochondrial interaction events in each eosinophil section showed that all of the three types of mitochondrial contacts were significantly higher within immature eosinophils compared to mature ones (Mitochondria-ER: immature = 16.80 ± 0.21%; mature = 7.18 ± 0.24%; Mitochondria-mitochondria: immature = 10.28 ± 0.30%; mature = 6.99 ± 0.32%; Mitochondria-granule: immature = 25.38 ± 0.62%; mature = 8.04 ± 0.42%; n (mitochondria) = 166 for immature and 151 for mature eosinophils; *p* < 0.0001 for all types of interaction) (Figures 5A–C). Remarkably, by evaluating the total number of mitochondria in physical contact with cytoplasmic granules in immature eosinophils, we found that 69% of these interactions were established with coreless granules in process of content filling while 31% were in contact with cored granules (Figure 4I, Figure 5D). On the other hand, the distribution of these two types of granules in the cytoplasm did not vary (Figures 5E,F).

Because this mitochondria-granules interaction particularly drew our attention, we performed multivariate logistic regression analyses to evaluate the proximity of mitochondria with other 7 cell compartments (plasma membrane, nucleus, endoplasmic reticulum, Golgi apparatus, mitochondria, immature coreless granules, and mature cored granules) with independent variables being used to determine the influence of these multiple factors at the same time. Our data confirmed that a greater number of mitochondria in close proximity to coreless granules (OR: 1.030, *p* = 0.035) or other mitochondria (OR: 1.014, *p* = 0.021) is associated with an increased chance of a sample being of immature eosinophils while a greater number of the mitochondria in close proximity to cored granules (OR: 0.892, *p* = 0.002) is associated with an increased chance of the sample being of mature eosinophils (*n* = 503 mitochondria from immature and 166 from mature eosinophils) with a pseudo *R* ^2^ (Nagelkerke) of 0.77 and a predictive accuracy of 91.2%. Altogether, our data point out that the mitochondrion-granule interaction is not a random phenomenon.

**FIGURE 6:**
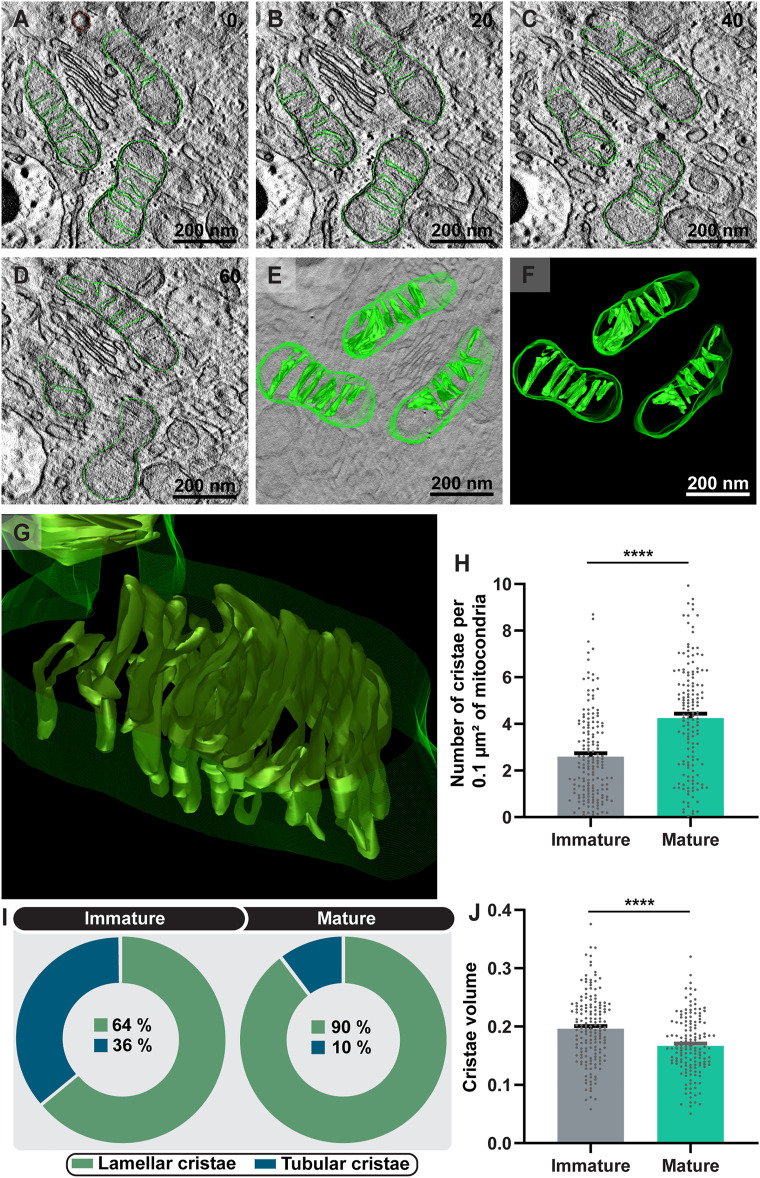
Eosinophilopoiesis leads to increased formation of mitochondrial cristae and cristae remodeling. **(A–G)** Representative 4 nm-digital slices of a tomogram showing mitochondria with lamellar cristae, the most common shape of cristae found within developing mouse eosinophils. Mitochondria boundaries and internal membranes in consecutive tomogram slices were manually contoured in green and computer-based 3D models were generated **(F)**. In **(G)**, the aspect of lamellar cristae is shown in higher magnification. Virtual slices were extracted from 3D reconstructions of a 200 nm eosinophil section analyzed by fully automated electron tomography at 200 kV. The numbers on the upper right corner indicate the slice number through the tomographic volume. Both the total number of cristae **(H)** and the lamellar type **(I)** significantly increased in concert with maturation. **(J)** The cristae volume was reduced in mature eosinophils. Samples were prepared for TEM and electron tomography as before (Melo et al., 2005). Quantitative analyses were performed using Fiji software. Scattered dots represent 0.1 µm^2^ of the mitochondrial area in **(H)** and single mitochondria data in **(J)**. A total of 317 mitochondria (166 from immature and 151 from mature) were evaluated at high resolution within 48 eosinophils (28 immature and 20 mature) collected from at least three independent cell cultures. Results are expressed as means ± SEM. (****p < 0.0001).

Third, we asked if the mitochondrial cristae remodeling would be involved in the process of eosinophil differentiation. The ultrastructural morphology of the mitochondrial cristae was then analyzed in terms of numbers, morphology, and volume. Two patterns of mitochondrial cristae were found: lamellar and tubular, with mitochondria exhibiting predominantly lamellar cristae in both immature and mature eosinophils as shown by both conventional and electron tomography (Figures 6A–G). TEM quantitative analyses showed that the total number of mitochondrial cristae significantly increased in parallel to the eosinophil development (immature: 2.60 ± 0.14 versus mature: 4.25 ± 0.19 (mean ± SEM); n (mitochondria) = 166 for immature and 151 for mature eosinophils; *p* < 0.0001) (Figure 6H). Moreover, the number of mitochondria exhibiting lamellar cristae also increased with the process of maturation, that is, as the eosinophil matures cristae reshapes to the lamellar morphology (Figure 6I). In contrast, the cristae volume was reduced in mature eosinophils (immature: 0.196 ± 0.005 versus mature: 0.166 ± 0.004 (mean ± SEM); n (mitochondria) = 166 for immature and 151 for mature eosinophils; *p* < 0.0001) (Figure 6J).

### Mitochondrial Dynamics During Inflammatory Diseases

Next, we wondered if the mitochondrial dynamics would change ultrastructurally in mature eosinophils participating in inflammatory diseases. We then evaluated the mitochondrial ultrastructure in well-established mouse models of allergic inflammation/asthma (Serra et al., 2012) and IAV (Samarasinghe et al., 2017; LeMessurier et al., 2020) and parasite (schistosomiasis mansoni) (Amaral et al., 2017; Dias et al., 2018) infections, all conditions characterized by eosinophil recruitment and activation. Mitochondria were studied *in situ* in eosinophils from the lung parenchyma (allergic inflammation), large intestine (schistosomiasis mansoni), and in BALs (IAV infection) after cell fixation and processing for TEM following the same protocols that we have been using to conduct pathological analyses of human eosinophils in tissues and cell suspensions, which enable optimal morphology (Melo et al., 2022).

**FIGURE 7:**
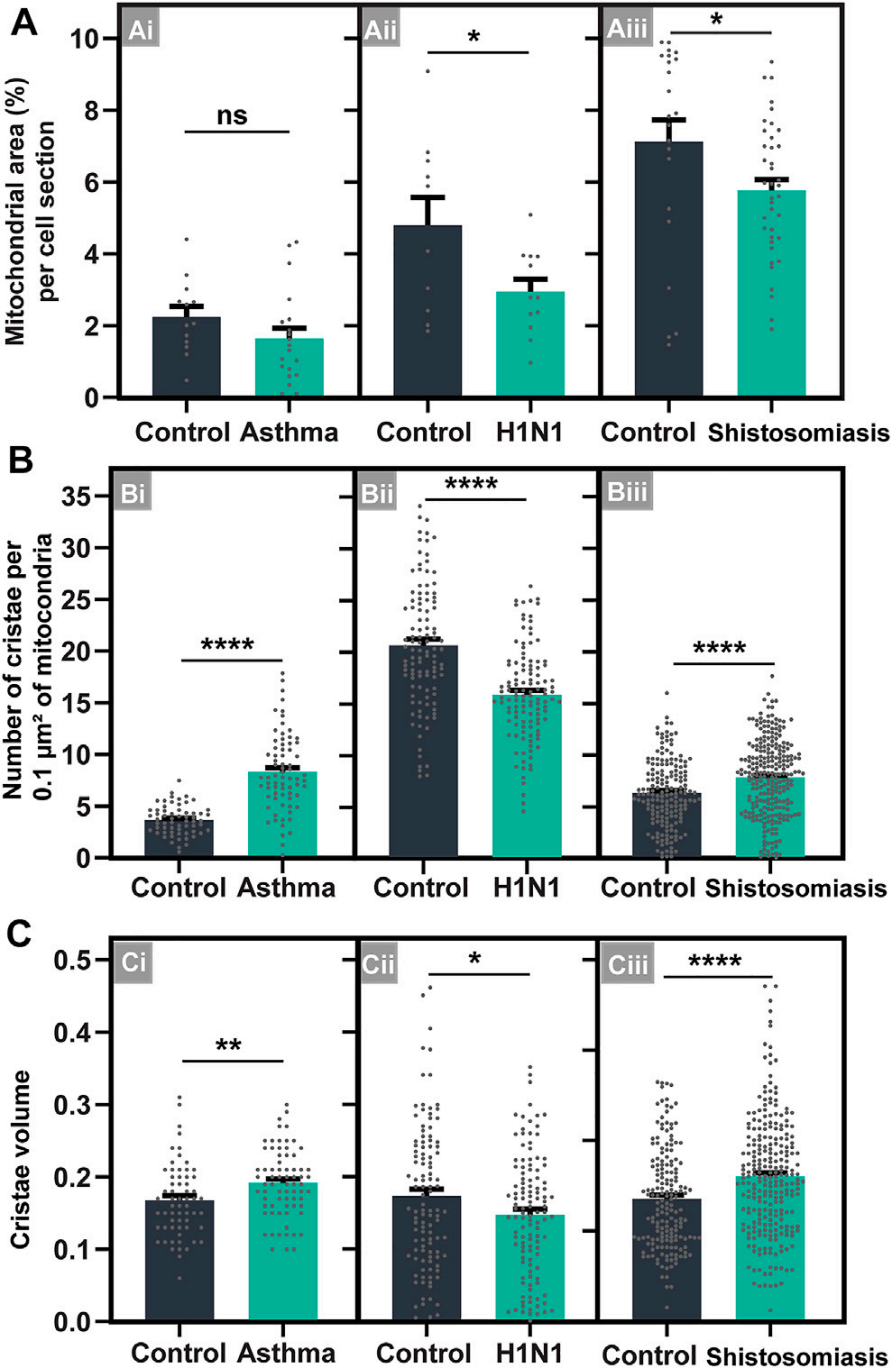
Inflammatory diseases induce consistent mitochondrial cristae remodeling within infiltrating tissue eosinophils in mice. **(A)** The total mitochondrial area in the eosinophil cytoplasm did not change in asthma (Ai) or is reduced in H1N1 IAV infection (Aii) and schistosomiasis mansoni (Aiii), in response to the inflammatory diseases. **(B,C)** The total number **(B)** and volume **(C)** of cristae is increased in both asthma (Bi and Ci) and schistosomiasis (Biii and Ciii), but not in the H1N1 IAV infection (Bii and Cii). Samples were prepared for TEM and quantitative analyses performed using Fiji software. Scattered dots represent single-cell data in **(A)**; 0.1 µm^2^ of the mitochondrial area in **(B)**; and single mitochondria data in **(C)**. A total of 795 mitochondria were evaluated at high resolution within 116 cells collected from at least five independent experiments for each condition. Cells were prepared for TEM. Results are expressed as means ± SEM (*p < 0.05; **p < 0.01; ****p < 0.0001; ns (not significant) *p* > 0.05).

Compared to control eosinophils from the same organ/tissue/condition, the cytoplasmic area occupied by mitochondria within eosinophils did not change in allergic inflammation and it was reduced in response to both viral and parasitic infections (Figures 7Ai–Aiii; See also Supplementary Figure S3). Means ± SEM (*n* = cell sections) for cytoplasmic area occupied by mitochondria are: 1) asthma: 2.24 ± 0.28 (control, *n* = 13) *versus* 1.64 ± 0.28 (treated, *n* = 20); *p* = 0.16; 2) schistosomiasis: 7.12 ± 0.61 (control, *n* = 22) *versus* 5.76 ± 0.3 (infected, *n* = 39); *p* < 0.05; 3) H1N1 IAV infection: 4.79 ± 0.77 (control, *n* = 10) *versus* 2.94 ± 0.34 (infected, *n* = 12); *p* < 0.05.

On the other hand, our imaging analyses at high resolution captured a significant increase in the numbers (Figures 7Bi–Biii) and volume (Figures 7Ci–Ciii) of mitochondrial cristae in response to allergic inflammation and schistosomiasis, but not to the H1N1 IAV infection, which lead to the reduction of both cristae numbers and volume. Means ± SEM (*n* = number of mitochondria) for cristae numbers are: 1) asthma: 3.62 ± 0.36 (control, *n* = 62) and 7.82 ± 1.02 (treated, *n* = 72); *p* < 0.0001; 2) schistosomiasis: 6.36 ± 0.37 (control, *n* = 175) and 7.5 ± 0.31 (infected, *n* = 257); *p* < 0.0001; 3) H1N1 IAV infection: 20.36 ± 1.46 (control, *n* = 110) and 15.32 ± 1.17 (infected, *n* = 119); *p* < 0.0001. Means ± SEM (n = number of mitochondria) for cristae volume are: 1) asthma: 0.17 ± 0.006 (control, *n* = 62) and 0.19 ± 0.005 (treated, *n* = 72); *p* = 0.006; 2) schistosomiasis: 0.14 ± 0.004 (control, *n* = 175) and 0.16 ± 0.004 (infected, *n* = 257); *p* < 0.001; 3) H1N1 IAV infection: 0.17 ± 0.009 (control, *n* = 110) and 0.14 ± 0.007 (infected, *n* = 119); *p* = 0.032).

**FIGURE 8:**
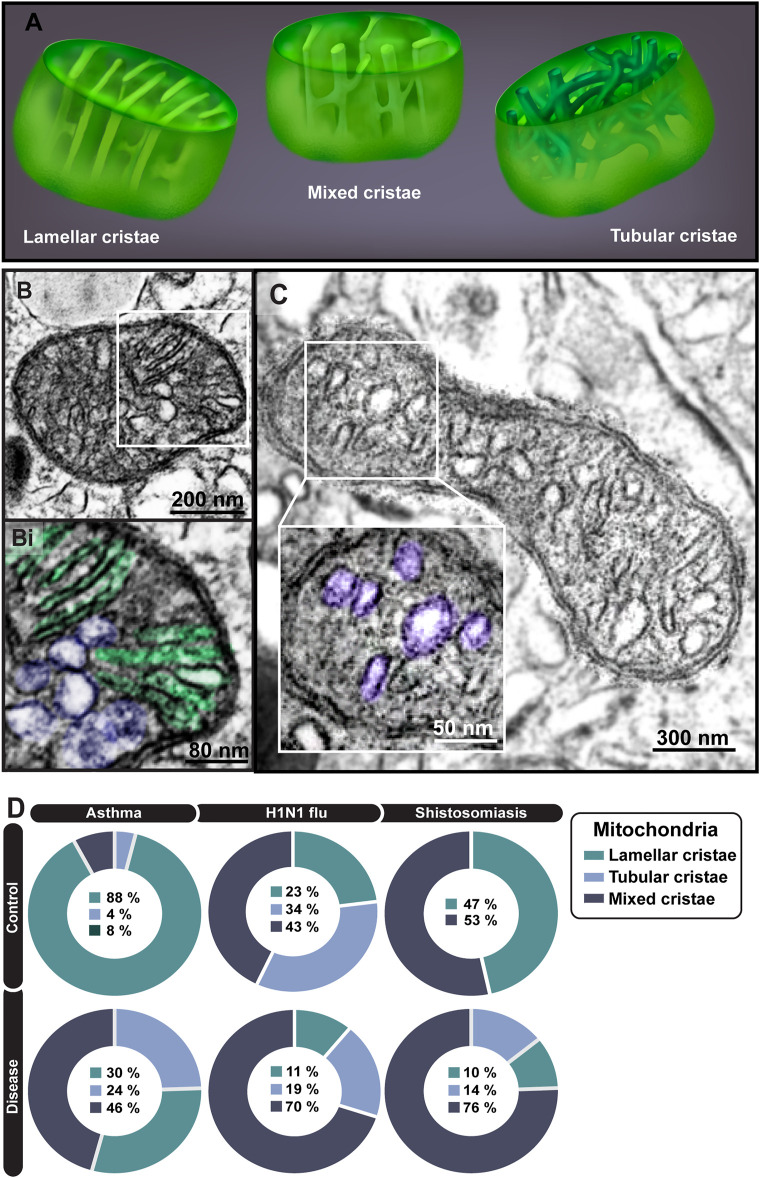
Ultrastructural aspects of mitochondrial cristae within mouse eosinophils. **(A)** Illustration depicting the three types of cristae—lamellar, tubular, and mixed. In **(B)**, a representative mitochondrion with both lamellar (highlighted in green in Bi) and tubular (highlighted in purple in Bi) cristae while **(C)** shows a mitochondrion with predominantly tubular cristae (colored in purple in Ci). **(D)** Cristae remodeling is observed in all diseases in inflammatory eosinophils compared to resident ones. A total of 795 mitochondria were evaluated at high resolution within 116 cells collected from at least five independent experiments for each condition. Cells were prepared for TEM.

When in the tissue microenvironment, mitochondria within eosinophils formed mixed cristae, a morphological pattern characterized by tubular and lamellar cristae within the same mitochondrion (Figures 8A–C), not observed in developing eosinophils. Quantitative TEM showed that, in general, eosinophils participating in inflammatory responses reduced the numbers of lamellar cristae and increased the numbers of mixed cristae in their population of mitochondria (Figure 8D). However, the proportions of mitochondria containing only lamellar or tubular, or mixed cristae changed depending on the cell microenviroment/disease (Figure 8D).

**FIGURE 9:**
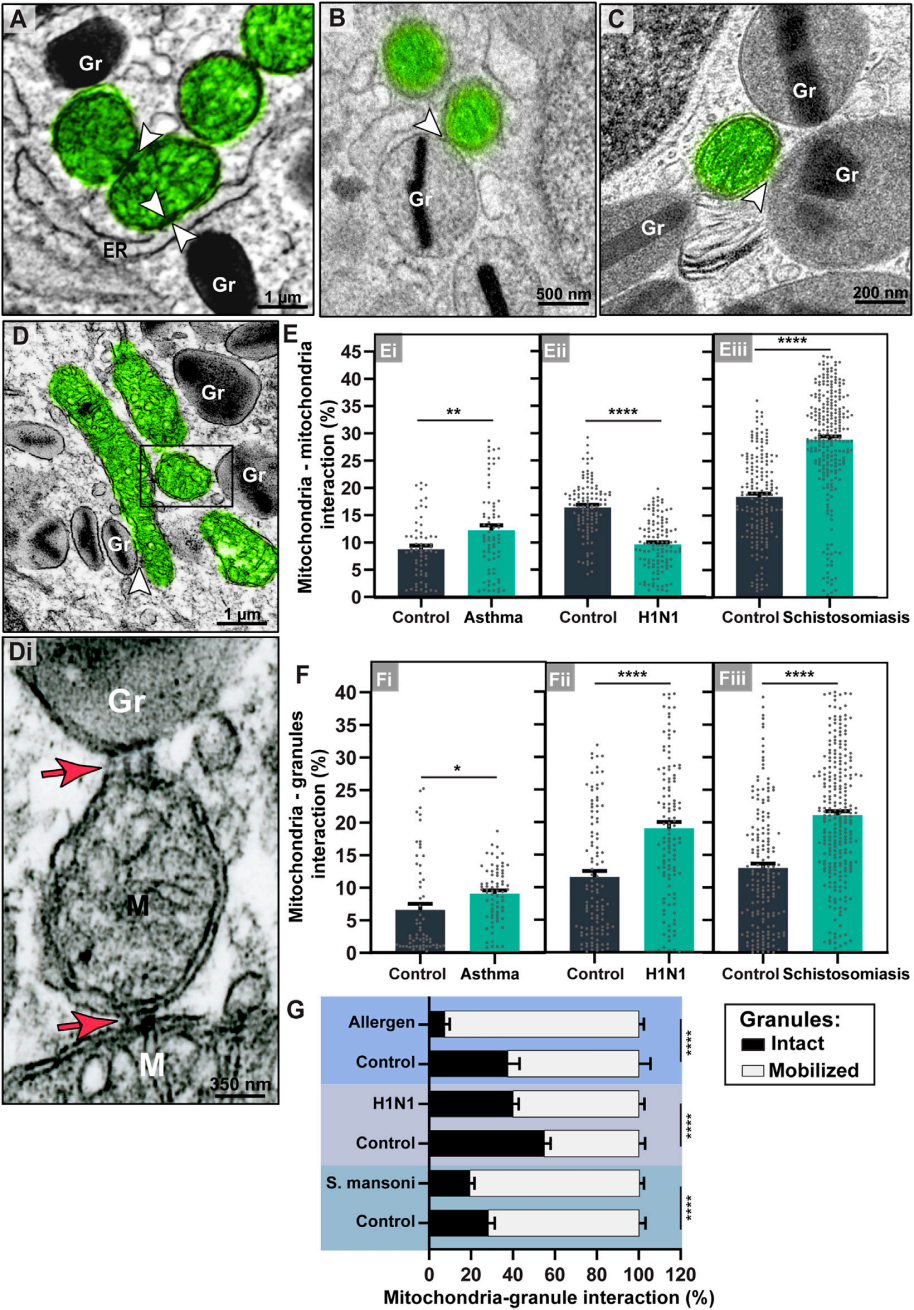
Mitochondrial inter-organelle contact sites within tissue mouse eosinophils. **(A–D)** Representative electron micrographs showing mitochondrial interactions (arrowheads and boxed area) with the ER, other mitochondria, or secretory granules (Gr) within inflammatory eosinophils recruited by schistosomiasis mansoni (A and B, large intestine), asthma (C, lung), and H1N1 IAV infection (D, BAL). The boxed area in **(D)** is shown in (Di). Note the presence of tethers (red arrows) at the interface mitochondrion-mitochondrion (M) and mitochondrion-secretory granule (Gr). (Ei-Eiii) Mitochondria-mitochondria contact sites increased within eosinophils in asthma and schistosomiasis but not in response to H1N1 IAV infection. (Fi-Fiii) All inflammatory diseases led to a significant increase of mitochondria-granules interactions, notably with mobilized granules as shown in **(G)**. Mitochondria were colored in green in **(A–D)**. Scattered dots in **(E,F)** represent single mitochondria data. A total of 795 mitochondria were evaluated at high resolution within 116 cells collected from at least five independent experiments for each condition. Samples were prepared for TEM. Results are expressed as means ± SEM (*p < 0.05; **p < 0.01; ****p < 0.0001).

Finally, we sought for mitochondrial interactions within inflammatory eosinophils (Figures 9A–D). By enumerating inter-organelle membrane contacts, we found that while mitochondria-ER contacts as seen in Figure 9A were not significantly increased during inflammatory responses in all diseases (*p* > 0.05), mitochondria-mitochondria interactions increased in asthma (Means ± SEM (n = mitochondria) of 8.74 ± 0.69% (control, *n* = 62) *versus* 12.22 ± 0.93% (treated, n = 72); *p =* 0.004; (Figure 9Ei) and schistosomiasis mansoni (means ± SEM (*n* = mitochondria) of 18.44 ± 0.61% (control, *n* = 172) *versus* 28.87 ± 0.62 (infected, *n* = 257); *p* < 0.0001); (Figure 9Eiii); and reduced in the H1N1 IAV infection (means ± SEM (n = mitochondria) of 16.40 ± 0.48% (control, *n* = 110) *versus* 9.60 ± 0.43% (infected, *n* = 119); *p* < 0.0001 (Figure 9Eii).

Remarkably, mitochondria contact sites with secretory granules significantly increased in all infections (Figure 9F). The means ± SEM (n = mitochondria) are: 1) Asthma model (6.62 ± 0.93% (control, *n* = 62) *versus* 9.11 ± 0.49% (treated, *n* = 72); *p* = 0.01; 2) H1N1 IAV model (11.58 ± 0.89% (control, *n* = 110) *versus* 19.04 ± 0.96% (infected, *n* = 119; *p* < 0.0001; 3) shistosomiasis model (12.97 ± 0.71% (control, *n* = 172) *versus* 21.10 ± 0.60% (infected, n = 257); *p* < 0.0001). These mitochondria-granule associations involved not only membrane contacts, frequently seen as areas with increased electron density, but also tethers (narrow “projections” from the mitochondrial outer membrane), as previously described (Csordás et al., 2006) (Figures 9D,Di). These links were also shown at the interface mitochondria-mitochondria (Figure 9Di).

To get more insights into this novel identified ability of eosinophil mitochondria to interact with granules, we then wondered if these granules exhibited signs of mobilization, that is, signs indicative that their contents were in process of release. By our experience, the specific granules of mouse eosinophils show a more elliptical/elongated morphology and are less prone to undergo swelling than specific granules of human eosinophils (Melo et al., 2022). The occurrence of this event, in addition to matrix coarsening and content disarrangement, in the absence of granule fusions, is an important sign that the granules are being responsive to the tissue microenvironment with mobilization of their contents through piecemeal degranulation (Shamri et al., 2012; Dias et al., 2018; Melo et al., 2022). As expected, infiltrating inflammatory eosinophils had higher rate of mobilized secretory granules compared to control eosinophils (Supplementary Figure S4). We found that the mitochondria-granule interactions were significantly increased with mobilized granules (Figure 9G) and that such interaction does not seem to be occurring as a result of the granule distribution in the cytoplasm (Supplementary Figure S4).

Altogether, our results show the mitochondrial population of mouse eosinophils is influenced by the surrounding tissue microenvironment and can respond to inflammatory conditions with cristae remodeling and inter-organelle contacts.

## Discussion

TEM is the technique of choice to visualize in detail the mitochondria architecture including their size, cristae shapes, contacts with other organelles, and morphological changes in the cell cytoplasm during different conditions [reviewed in (Duranova et al., 2020; Glancy, 2020)]. Here, we provide a systematic study of the mitochondrial ultrastructure in mouse eosinophils developing in cultures as well as recruited by inflammatory diseases. In the present work, we found that 1) the mitochondrial population significantly decreases during eosinophil differentiation and autophagy is likely playing a role in such reduction; 2) as the cell matures, mitochondrial cristae increase in number and reshapes to lamellar morphology while their volume is reduced by the end of maturation; 3) inflammation does not affect (asthma) or induces reduction (H1N1 IAV and parasitic infections) of the mitochondrial mass while cristae reshaping is a consistent event in all inflammatory conditions; 4) cristae numbers and volume within individual mitochondria can increase or decrease in response to inflammatory diseases depending on the microenvironment; and 5) mitochondria interact with other organelles, notably with secretory granules during both eosinophil differentiation and inflammation-induced cell activation.

In general, mature cells of the immune system, including eosinophils (Peachman et al., 2001), are deemed to have few numbers of mitochondria in the cytoplasm (Campello and Scorrano, 2010). Here, we show for the first time that the process of eosinoophilopoiesis impacts not only the entire mitochondrial population but also the size of single mitochondria (Figure 2) and that mitophagy is likely playing a role in mitochondrial clearance in immature cells (Figure 3). Differentiation-induced mitophagy has been best demonstrated during the development of red blood cells and, in this case, it is not considered a mechanism involved in mitochondrial quality control, but rather a programmed mechanism operating to remove the mitochondrial population [reviewed in (Mortensen et al., 2010)]. A similar mechanism might be operating in eosinophils and potentially in other leukocytes, but while mitochondria are completely removed from erythrocytes, a population of mitochondria remains in eosinophils. As captured by our high-resolution analyses, this smaller mitochondrial population within mature eosinophils has increased numbers of cristae and more cristae with lamellar morphology than mitochondria from immature eosinophils, thus denoting the occurrence of a clear cristae remodeling during the process of eosinophil differentiation.

Mitochondrial cristae are crucial for mitochondria. These specialized compartments of the mitochondrial internal membrane are closely related to the mitochondria respiratory performance because specific chemical reactions and different complexes of proteins are assembled in their extent. It is recognized that the shape, number, volume, and dimensions of the mitochondrial cristae are directly correlated with metabolic adaptation [reviewed in (Cogliati et al., 2016; Joubert and Puff, 2021)]. Thus, the finely balanced network of signaling pathways involved in the eosinophil development (from multipotent progenitors to terminally differentiated cells), which encompasses important cytokines such as IL-5 (Maltby et al., 2013) might be also modulating the population of mitochondria in a “preparation” for a different metabolic situation as circulating and mucosal resident cells. By the end of eosinophil development, there is a reduction in the cristae volume, which may indicate low metabolic activity at this stage while once in the tissue microenvironment, the volume increases or decreases depending on the disease condition. In fact, a study conducted with human peripheral blood eosinophils indicates that these cells are “metabolically flexible” with high adaptability to distinct demands of energy (Porter et al., 2018), which is in accord with our ultrastructural data. However, it is important to highlight that eosinophil bioenergetics, in both humans and mice, is still poorly understood.

The life cycle of the eosinophil is divided into bone marrow, blood, and tissue phases. Our ultrastructural findings revealed that once eosinophils are infiltrated in tissue inflammatory sites their mass of mitochondria did not change (asthma) (Figure 7Ai) or can be reduced (both infectious diseases) (Figures 7Aii,Aiii) while their cristae undergo remodeling (Figure 8). All inflammatory diseases led to changes in the cristae shape. Overall, in response to the inflammatory milieu, the numbers of lamellar cristae decreased with a parallel increase of mixed cristae, a morphological pattern characterized by both lamellar and tubular cristae, observed here just in mitochondria from tissue eosinophils (Figure 8). However, the proportions of mitochondria with lamellar, tubular, and mixed cristae vary depending on the microenvironment, thus, indicating that the mitochondria dynamics is responsive to the surrounding milieu. Our data are in accord with a previous work showing structural changes of mitochondrial cristae associated with activated leukocytes (Buck et al., 2016). While we cannot indicate which are the signaling pathways underlying inflammation-driven cristae remodeling, our findings implicate eosinophil inflammatory responses with mitochondrial dynamics. How mitochondrial cristae reshaping within eosinophils influences its immune functions is another open question to be addressed in future studies.

The mitochondrial behavior within eosinophils recruited by the IAV infection particularly drew our attention because several pieces of evidence show that H1N1 IAV target mitochondrial-dependent mechanisms [reviewed in (Cervantes-Silva et al., 2021)]. In infected epithelial cells, IAV leads to alterations of the mitochondrial dynamics and both intensified mitochondrial fission (Yoshizumi et al., 2014) or fusion/elongation (Pila-Castellanos et al., 2021) have been associated with this infection. In eosinophils, by measuring different stages of mitochondrial respiration, it was demonstrated that eosinophil respiration is significantly reduced following IAV exposure (LeMessurier et al., 2020). Our present findings are in accord and extend these observations showing that the lower respiration is probably due to a reduction of the mitochondrial mass, cristae numbers, and cristae volume which, as noted above are interlinked with the cell respiratory performance (Cogliati et al., 2016).

Mitochondria inter-organelle interactions as a mode to regulate cell functions have been a subject of great interest. While TEM has the limitation of relying on static observations, it enables distinguishing between contiguous membranes and accurate quantification of mitochondrial morphology and interactions (Picard et al., 2013). One of the most recognized of these mitochondrial interactions is with the ER within different cell types (Csordás et al., 2006), including epithelial cells in the context of lung diseases [reviewed in (Bruno and Anathy, 2021)] and immune cells [reviewed in (Angajala et al., 2018)]. For example, in T cells, such interaction enables metabolic reprogramming necessary for immune responses (Bantug et al., 2018). In eosinophils, mitochondria interactions are poorly understood. Here, we identified that during eosinophil differentiation events of mitochondria interconnectivity with other organelles (mitochondria-ER, mitochondria-mitochondria, and mitochondria-granules) are significantly higher in immature eosinophils compared to mature cells (Figures 4, 5) when developing in cultures.

In inflammatory tissue eosinophils, mitochondria-mitochondria interactions increase in all conditions except in the H1N1 IAV infection whereas mitochondria-ER interaction was not amplified likely because the peripheral ER in mature eosinophils is not a prominent organelle (Melo et al., 2022). While mitochondria-mitochondria interaction was an expected event since mitochondria work as a team exchanging matrix proteins and are able to fuse with each other (Liu et al., 2009), membrane contacts of mitochondria with secretory granules as well as structural links at the interface mitochondria-granules (Figure 9Di) were surprisingly detected (Figure 9). During differentiation, mitochondria contact more granules in process of filling (coreless) than mature (cored) granules. Inflammation directs mitochondria to contact more granules in process of content mobilization than intact granules (Figure 7). These findings are important because provide insights into potential mitochondrial interference/regulation with processes of eosinophil granulogenesis and secretion underlying inflammatory responses. It is clear that mitochondria have functions that go beyond cell respiration and the role of these organelles interrelated with the immune machinery is starting to emerge.

In conclusion, we identified that eosinophilopoiesis leads to a significant reduction of the mitochondrial population and that both differentiation and inflammation-induced activation interfere with the mitochondrial dynamics within mouse eosinophils. The resulting increased cristae remodeling and inter-organelle contacts, including mitochondria-secretory granules interactions, may potentially influence eosinophil immune responses. The understanding of how mitochondrial dynamics contribute to eosinophil immune functions is an open interesting field to be explored.

## Data Availability

The raw data supporting the conclusions of this article will be made available by the authors, without undue reservation.
