# Supplementary material for: Mitochondrial Population in Mouse Eosinophils: Ultrastructural Dynamics in Cell Differentiation and Inflammatory Diseases
Source: Front Cell Dev Biol. 2022 Mar 21;10:836755. doi: 10.3389/fcell.2022.836755 (PMC8979069; doi:10.3389/fcell.2022.836755)
Supplement: Supplementary file 1 [file Image4.pdf]

Supplementary Material

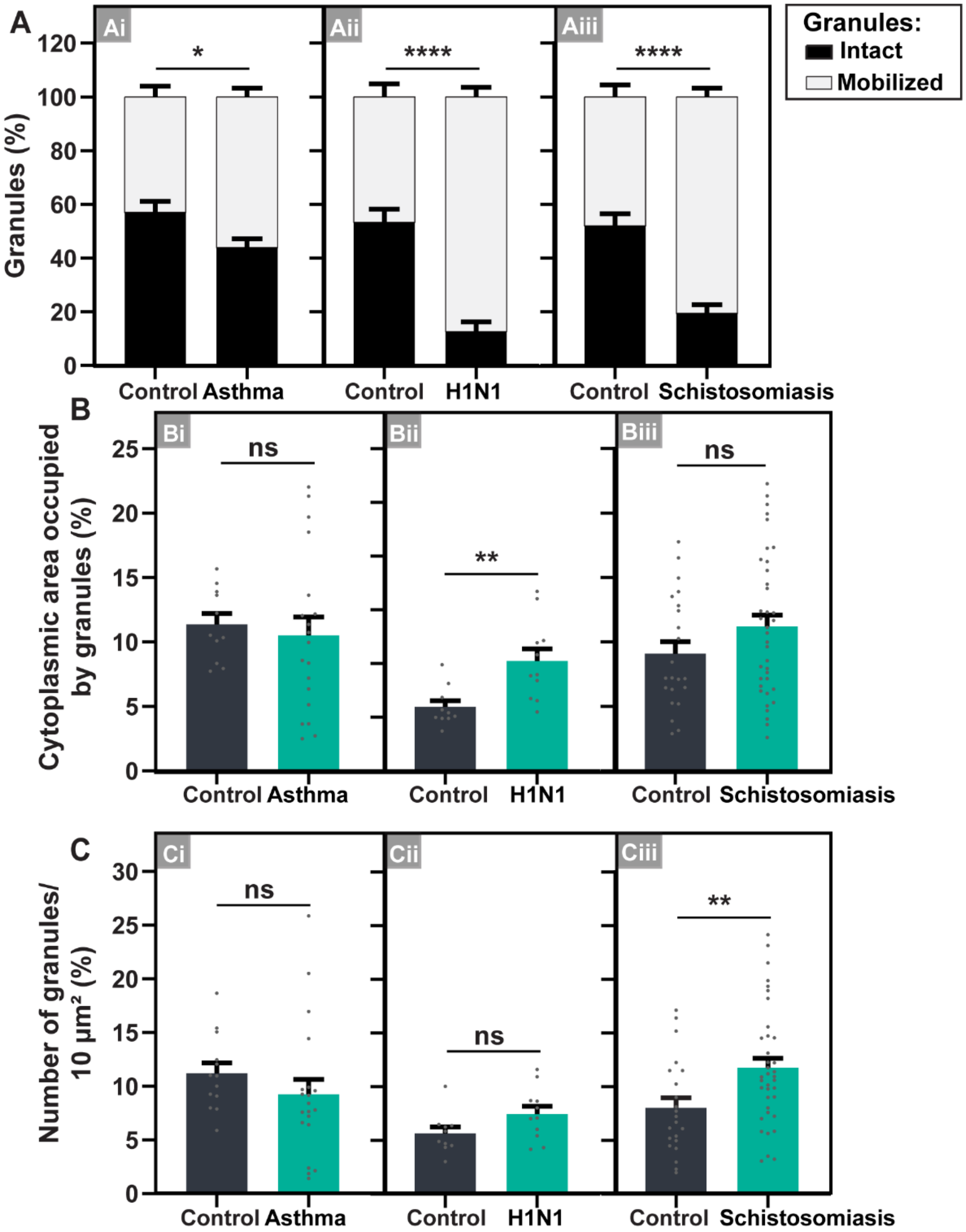

Supplementary Fig. 4. Quantitative analyses of secretory granules in mouse eosinophils during different diseases. A total of 2,183 granules were counted within 116 cells from at least five independent experiments for each condition. Scattered dots in B and C represent single cell sections. Results are expressed as means ± SEM (**P*<0.05; ***P*<0.01; *****P*<0.0001; ns (not significant) *P*>0.05).
