# Supplementary material for: Mitochondrial Population in Mouse Eosinophils: Ultrastructural Dynamics in Cell Differentiation and Inflammatory Diseases
Source: Front Cell Dev Biol. 2022 Mar 21;10:836755. doi: 10.3389/fcell.2022.836755 (PMC8979069; doi:10.3389/fcell.2022.836755)
Supplement: Supplementary file 2 [file Image2.pdf]

Supplementary Material

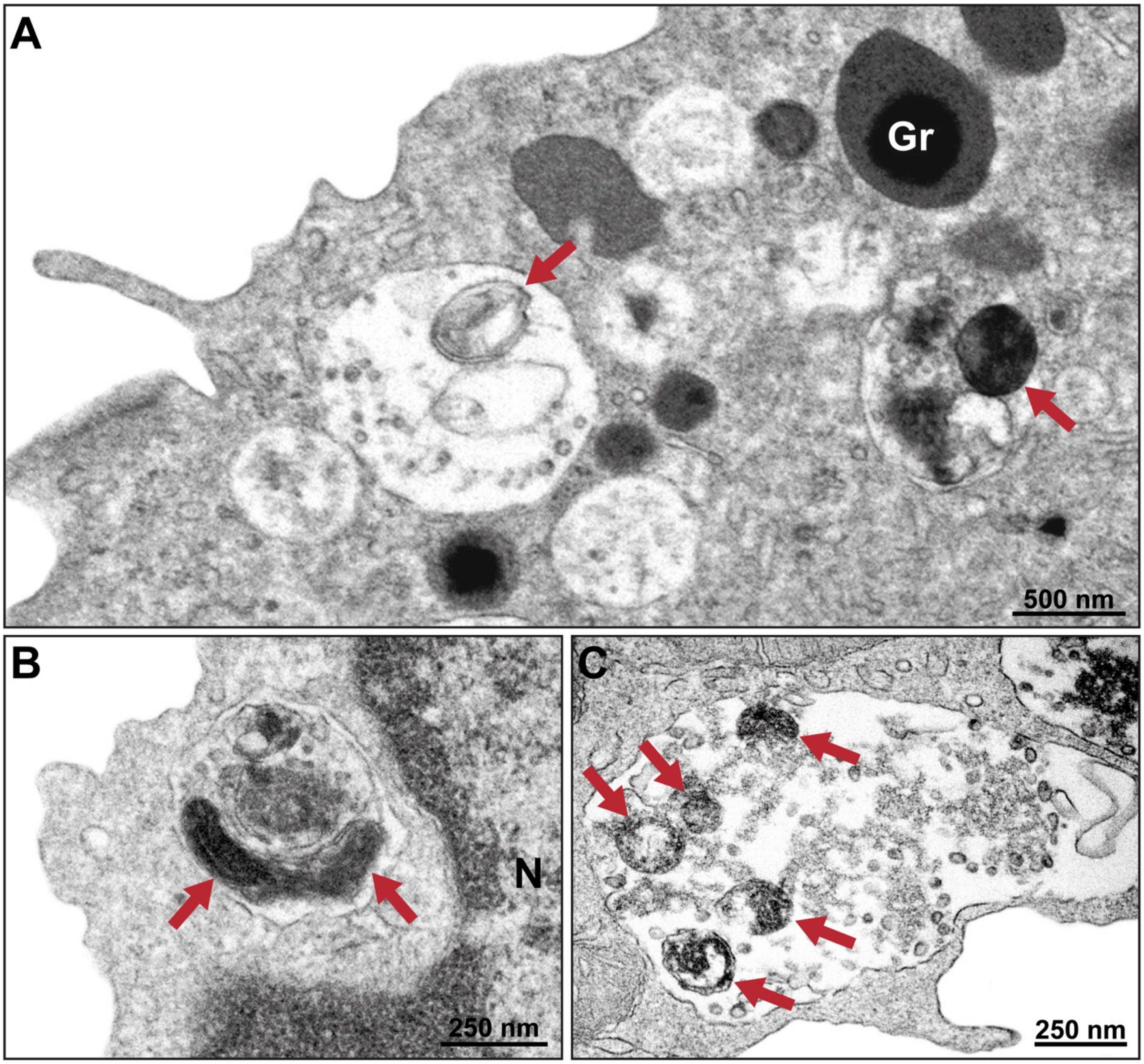

Supplementary Fig. 2. Electron micrographs from developing mouse eosinophils in cultures showing autophagic vacuoles containing degenerating mitochondria (arrows) in the lumen. Cells were prepared for transmission electron microscopy as described in material and methods.
