# Supplementary material for: Mitochondrial Population in Mouse Eosinophils: Ultrastructural Dynamics in Cell Differentiation and Inflammatory Diseases
Source: Front Cell Dev Biol. 2022 Mar 21;10:836755. doi: 10.3389/fcell.2022.836755 (PMC8979069; doi:10.3389/fcell.2022.836755)
Supplement: Supplementary file 3 [file Image3.pdf]

# *Supplementary Material*

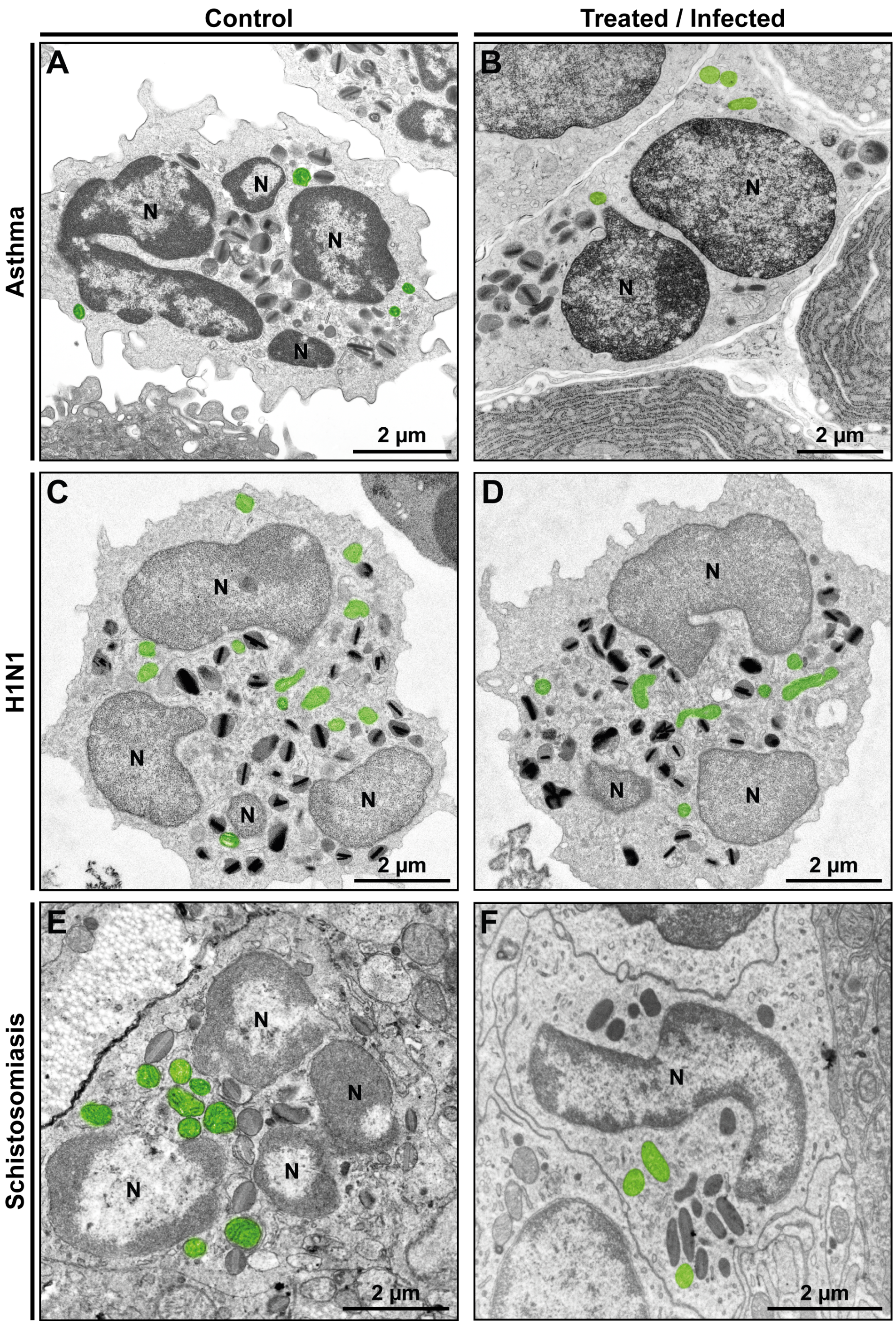


**Supplementary Fig. 3.** Representative electron micrographs of eosinophils in three experimental mouse models of diseases showing mitochondrial profiles highlighted in green. (A-B) Lung eosinophils from control and OVA-sensitized mice. (C-D) BAL eosinophils from noninfected and pH1N1 influenza virus-infected mice. (E-F) Intestinal eosinophils from noninfected and *Schistosoma mansoni*-infected mice. Models described as in Material and Methods. N, nucleus.
