# Supplementary material for: Mitochondrial Population in Mouse Eosinophils: Ultrastructural Dynamics in Cell Differentiation and Inflammatory Diseases
Source: Front Cell Dev Biol. 2022 Mar 21;10:836755. doi: 10.3389/fcell.2022.836755 (PMC8979069; doi:10.3389/fcell.2022.836755)
Supplement: Supplementary file 4 [file Image1.pdf]

Supplementary Material

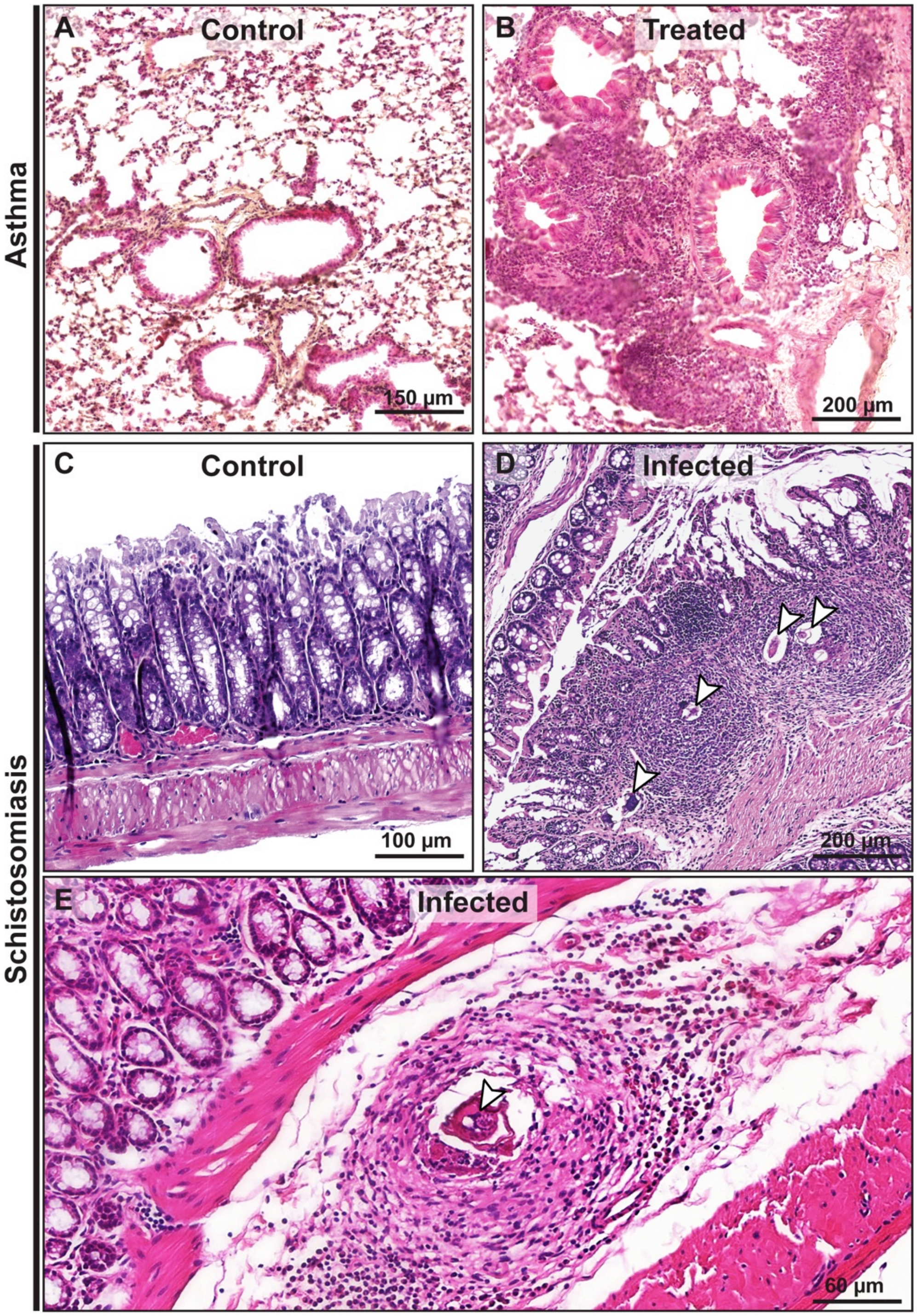

Supplementary Fig. 1. Histopathological views of the lung and large intestine from mouse models of asthma and schistosomiasis mansoni, respectively. (A-E) Both infections are characterized by intense eosinophilic inflammatory infiltrates. Note the formation of typical *Schistosoma* granulomas around parasite eggs (arrowheads) in the intestine. In (E), a granuloma is shown in higher magnification. Samples were stained with hematoxylin-eosin as before (Amaral et al., 2017).
